# Brazilian Corn Ethanol Coproducts for Pigs: Feeding Value and Blood Parameters

**DOI:** 10.3390/ani14142108

**Published:** 2024-07-19

**Authors:** Anderson Corassa, Igor Willian Wrobel Straub, Maicon Sbardella, Ana Paula Silva Ton, Charles Kiefer, Claudson Oliveira Brito, Vivian Luana Rothmund, Leonardo Willian Freitas

**Affiliations:** 1 Agrarian and Environmental Sciences Institute, Federal University of Mato Grosso, Sinop 78.550-000, MT, Brazil; igor_wrobel_straub@hotmail.com (I.W.W.S.); maicon.sbardella@ufmt.br (M.S.); ana.ton@ufmt.br (A.P.S.T.); vivianrothmund@gmail.com (V.L.R.); lwillianf86@gmail.com (L.W.F.); 2 Veterinary and Animal Science Department, Federal University of Mato Grosso do Sul, Campo Grande 79.070-900, MS, Brazil; charles.kiefer@ufms.br; 3 Animal Science Department, Federal University of Sergipe, São Cristovão 40.035-530, SE, Brazil; claudson@academico.ufs.br

**Keywords:** DDGS, digestibility, HPDDG, metabolizable energy, urea

## Abstract

**Simple Summary:**

The variability in the chemical composition of cereal ethanol coproducts is a limiting factor in the precise use of these ingredients in swine feed. The growth of the corn ethanol industry in Brazil has boosted the availability of diverse coproducts but still lacks proper nutritional characterization, which may differ from other places in the world. The purpose of this study was to determine the values of the net, digestible and metabolizable energy and digestibility coefficients of corn ethanol coproducts produced in Brazil and their effects on the nitrogen balance and blood parameters of pigs. Pigs fed diets with high-protein distiller’s dried grain and corn bran with solubles showed greater nitrogen retention efficiency than pigs fed distiller’s dried grains with solubles, while pigs fed diets containing corn bran with solubles had lower urea and higher blood triglycerides.

**Abstract:**

This study aimed to determine the values of net energy (NE), digestible energy (DE) and metabolizable energy (ME) and digestibility coefficients of corn ethanol coproducts produced in Brazil and their effects on the nitrogen balance and blood parameters of pigs. Ten barrows were housed in metabolic study cages for total collection and fed a reference diet (RD) or 800 g/kg RD + 200 g/kg of a coproduct of corn ethanol. Distiller’s dried grains with solubles (DDGS), corn bran with solubles (CBS), distiller’s dried grains (DDG) and high-protein distiller’s dried grain (HPDDG) were evaluated. The experimental design was randomized blocks with three repetitions per period, totaling six repetitions per diet. Diets containing the HPDDG had greater DE and ME than those containing CBS and DDGS and greater DE than those containing the DDG (*p* < 0.05). HPDDG, DDG, CBS and DDGS showed 4498, 3419, 3029 and 3335 kcal/kg DE; 4366, 3305, 2934 and 3214 kcal/kg ME; and 2515, 1938, 1649 and 1725 kcal/kg NE, respectively. Pigs fed diets containing HPDDG and CBS showed greater nitrogen retention efficiency than pigs fed DDGS (*p* < 0.05). Pigs fed diets containing HPDDG had higher blood urea levels than pigs fed CBS and RD, while triglyceride levels in animals that received the CBS diet were greater than those in animals that received all other diets. The HPDDG had the highest energy levels and the best digestibility coefficients. The chemical composition of coproducts influences the nitrogen balance and circulating levels of urea and triglycerides in pigs.

## 1. Introduction

Brazil is a major producer of sugarcane ethanol and has been significantly increasing corn ethanol production due to the installation of several industries, mainly in the central region of the country [1]. Variations in raw materials and in the processes of grinding, fermentation, drying, separation of fibers, inclusion of soluble compounds, use of additives and oil withdrawal are observed in mills to optimize the production of ethanol and coproducts according to specificity and interest [2,3]. Thus, diverse coproducts have been made available for use in pig feeding, suggesting that there is a difference in Brazilian coproducts [4], despite scarce information [5,6].

Due to the use of the corn starch fraction in the ethanol production process, the coproducts have higher concentrations of fiber, protein and lipids, which may alter the use of energy and nutrients by animals. Metabolizable energy variations from 3266 to 3696 and 2955 to 3899 kcal/kg were recorded by Kerr et al. [7] and Li et al. [8], respectively, while Dereck et al. [9] determined coefficients of variation of 25, 8, 36, 13 and 18% for ash, crude protein (CP), ether extract (EE), neutral detergent insoluble fiber (NDF) and lysine, respectively, when analyzing different ethanol coproducts. The presence of certain ingredients in the diet and its chemical composition can influence serum parameters and indicate metabolic and physiological functions [10]. Foods with high fiber content can reduce glucose levels in animals [11], while the protein level of the diet tends to influence the concentration of blood urea [12] and blood triglycerides [13].

Therefore, the hypothesis tested in this study was that there is a difference in the use of energy and chemical contents of diets and in nitrogen (N) retention and serum metabolic levels of pigs fed different corn ethanol coproducts. Thus, the objective of this study was to determine the values of digestible energy (DE), metabolizable energy (ME) and net energy (NE) and digestibility coefficients of corn ethanol coproducts produced in Brazil and their effects on the nitrogen balance and blood parameters of pigs.

## 2. Materials and Methods

The Ethics Committee on the Use of Farming Animals at the Federal University of Mato Grosso approved all procedures utilized in this research prior to implementation (protocol number 23108.017482/2022-58).

The experiment was carried out in the experimental warehouse of the Nonruminant Nutrition Research sector of the Institute of Agricultural and Environmental Sciences, Federal University of Mato Grosso (Sinop, Mato Grosso, Brazil, latitude −11°86′26″ and longitude −55°48′49″).

### 2.1. Animals, Experimental Design and Dietary Treatments

A metabolism study was carried out with total collection of feces and urine following the methodology described by Sakomura and Rostagno [14] using ten barrows of commercial strain (Agroceres PIC^®^, Rio Claro, Brazil), weighing 25.46 ± 3.5 kg, housed in metabolism cages (0.42 m × 1.35 m) with woven wire flooring, distributed in a randomized block according to individual weight, with repetition for each period, totaling six repetitions per diet, and maintained at 31 ± 3 °C and a relative humidity of 57–73% for the entire experiment.

Five diets were provided, with a reference diet (RD) based on corn and soybean meal formulated in accordance with the recommendations of Rostagno et al. [15] (for the nutrient requirements of 25–45 kg pigs) (Table 1) and four others composed of 800 g/kg RD and 200 g/kg of each corn ethanol coproduct. Therefore, the inputs to be evaluated (corn ethanol coproducts) comprised 20% of the diet and were combined with 80% of the reference diet to estimate the digestibility of these feeds following the methodology described by Sakomura and Rostagno [14].

The coproducts used were corn bran with solubles (CBS) and high-protein dried distiller’s grains (HPDDG; FS Bioenergia, Lucas do Rio Verde, MT, Brazil), dried distiller’s grains (DDG; Destilaria de Álcool Libra Ltd.a, São José do Rio Claro, MT, Brazil) and dried distiller’s grains with solubles (DDGS; Safras Indústria e Comercio de Biocombustíveis Ltd.a, Sorriso, MT, Brazil) (Table 2).

Five initial days were used to acclimatize the animals to the cages, and another four-day period was used to adapt them to the diets before each five-day period of total collection of feces and urine. Three periods of feeding and total collection were performed.

The diets were mixed with water at a 1:1 ratio (1 g of feed to 1 g of water) and provided twice a day with daily records of leftovers and food consumption. The lowest feed intake per unit of metabolic weight of the pigs was used as a supply marker for all animals in the total collection period [14].

Feces were collected in trays at the back of the cages, while urine was collected in collectors under the cages containing 20 mL of 6 N HCl [9]. The daily production of feces was recorded at 07:30 and 17:00, and that of urine was recorded at 17:00, with 200 g/kg frozen (−10 °C). After the collection period, the samples were homogenized into a single sample per animal.

### 2.2. Digestible Content and Nitrogen Balance

Samples of the diets, coproducts and feces were thawed, homogenized, weighed, registered and kept in a forced ventilation oven at 55 °C for 72 h for partial drying and subsequent analysis in duplicate for dry matter (DM, method 934.01) [16], CP (method 2001.11) [16], EE (method 945.38) [16], ash (method 923.03) [15] and NDF (method INCT-CA F-001/1) [17].

The OM was obtained as the difference between the DM and ash. The particle size (PS) was obtained by calculating the mean geometric diameter [18]. The urine samples were thawed and homogenized for total N analysis (method 2001.11) [16]. The gross energy (GE) values of the feces, urine, diets and coproducts were determined using a bomb calorimeter (Parr 6400 calorimeter, Parr Instruments Co., Moline, IL, USA).

The apparent total tract digestibility (ATTD), digestible nutrient contents and coefficients of diets with ethanol corn coproducts were determined by the ratio of consumption to excretion of each component per animal according to the methodology described by Sakomura and Rostagno [14].

The DE and ME values of the diets and coproducts were determined according to the equations proposed by Matterson et al. [19] (Equations (1) and (2)) and, for the NE, according to the equation proposed by Wu et al. [20] (Equation (3)). Once the DE and ME values were obtained, the ratios of DE:GE, ME:GE and NE:GE were estimated.

(1) $$\mathrm{DE}\;\mathrm{value}\;\mathrm{of}\;\mathrm{test}\;\mathrm{ingredient}\left(kcal/kg\right)=\mathrm{DE}\;\mathrm{reference}\;\mathrm{diet}+\frac{\left(\mathrm{DE}\;\mathrm{test}\;\mathrm{diet}-\mathrm{DE}\;\mathrm{reference}\;\mathrm{diet}\right)}{0.20}$$

(2) $$\mathrm{ME}\;\mathrm{value}\;\mathrm{of}\;\mathrm{test}\;\mathrm{ingredient}\left(kcal/kg\right)=\mathrm{ME}\;\mathrm{reference}\;\mathrm{diet}+\frac{\left(\mathrm{ME}\;\mathrm{test}\;\mathrm{diet}-M\;\mathrm{reference}\;\mathrm{diet}\right)}{0.20}$$

(3) NE=−1130.5+(0.727 × GE) + (23.86 × EE) − (10.83 × NDF)

The N balance was determined using the N intake present in the diets, the N excreted in the feces and urine of the pigs and the N retained (intake less excreted) [14].

### 2.3. Blood Parameters

On the last day of each collection period, 35 min after the morning feeding, a blood sample was collected via puncture of the external jugular vein in the neck of each pig using a 10 mL syringe and a 40 mm × 1.20 mm needle. Approximately 4 mL of blood was transferred to Eppendorf tubes with sodium fluoride for further analysis of glucose, and another 4 mL was transferred into Eppendorf tubes with a clot activator for further analysis of triglycerides and urea. The samples were centrifuged at 3000 rpm for 15 min to obtain serum, and the contents were transferred to cryovial tubes.

The levels of glucose, triglycerides and urea were determined with diagnostic kits (Labtest^®^ Diagnóstica S.A., Lagoa Santa, MG, Brazil) for Glucose PAP Liquiform Vet (GOD-Trinder), Triglicerides Liquiform Vet (Enzimático-Trinder) and Urea UV Liquiform Vet (Enzymatic UV) using a semiautomatic biochemical analyzer (model: Spectrum, brand: Celer Biotecnologia S.A., Belo Horizonte, MG, Brazil) according to the method of Verussa et al. [21].

### 2.4. Statistical Analysis

The experimental design consisted of randomized blocks that were repeated, with five diets (reference and four test diets) and three repetitions per period, totaling six repetitions per treatment, and each pig as the experimental unit. The following statistical model was used:

Yij=μ+Ci+Rj+εij.

where Yij = observations referring to the effect of source i by the number of repetitions j; μ = overall mean; Ci = coproduct (HPDDG, DDG, CBS or DDGS); Rj = number of repetitions; and εij = random error associated with each observation. Diets were considered fixed effects, while animals and periods were considered random effects. Means were generated by the PDIFF command of SAS (SAS Institute Inc., Cary, NC, USA), with adjustment for comparison by Tukey’s test. The data were subjected to analysis of variance, and *p*-values < 0.05 were considered significant.

## 3. Results

### 3.1. Digestible Contents and Nitrogen Balance

**Figure 1:**
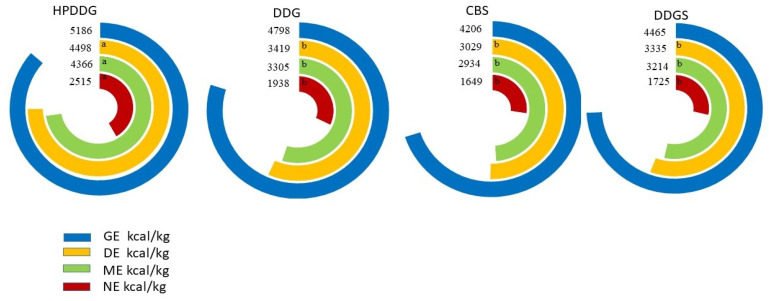
Energy values of the ethanol corn coproducts. HPDDG = high-protein distiller’s dried grain; DDG = distiller’s dried grains; CBS = corn bran with solubles; DDGS = distiller’s dried grains with solubles; GE = gross energy; DE = digestible energy; ME = metabolizable energy; NE = net energy. ^a,b^ Means followed by different letters on the line differ statistically at 5% probability according to the Tukey test.

Diets containing the HPDDG had greater DE and ME than those containing CBS and DDGS and greater DE than those containing the DDG (*p* < 0.05). HPDDG had greater DE:GE, ME:GE and NE:GE values than DDG and CBS (*p* < 0.05) and greater NE:GE values than DDGS (*p* < 0.05) (Table 3), which generated greater DE, ME and NE values than the other coproducts (*p* < 0.05) (Figure 1). There was no difference between DDG, CBS and DDGS coproducts for the DE, ME and NE values.

Diets containing HPDDG showed higher values of digestible contents compared to those containing CBS and DDGS (OM and NDF, *p* < 0.05), DDG, CBS and DDGS (CP, *p* < 0.05) and DDG and DDGS (EE, *p* < 0.05) (Table 4). The digestibility of the diets containing DDGS was similar to that of the diets containing DDG and CBS (OM), CBS (CP and NDF) and DDG (EE). The values of the diets containing HPDDG showed greater ATTD in relation to those containing DDG and DDGS (OM and EE, *p* < 0.05) and DDG, CBS and DDGS (NDF, *p* < 0.05), whereas the diets containing DDG showed greater ATTD values for NDF than those containing CBS (*p* < 0.05). The digestible DM, ash and ATTD of the DM, CP and ash did not differ among the diets.

In terms of nitrogen balance, the diets containing HPDDG had greater effects than the diets containing CBS and DDGS (N intake and N retained, *p* < 0.05) or CBS (N excreted, *p* < 0.05) and did not differ from the diet containing DDG (N intake and N retained) or DDG and DDGS (Table 5). The results of N intake, N excreted and N retained by the animals did not differ with the inclusion of DDG, CBS or DDGS coproducts in the diets. The diets did not influence the amounts of N in the feces or N in the urine. The N retention efficiency of pigs fed diets containing HPDDG and CBS was greater than that of pigs fed diets containing DDGS (*p* < 0.05).

### 3.2. Blood Parameters

There was no difference in glucose levels between the groups (Table 6). Pigs fed diets containing HPDDG had higher blood urea levels than pigs fed CBS and RD (*p* < 0.05). Diets containing RD, DDG, CBS and DDGS did not differ in urea yield. Blood triglyceride levels in animals that received the CBS diet were greater than those in animals that received all other diets (*p* < 0.05), while the blood triglyceride levels in animals that received the diets containing HPDDG and DDGS did not differ and were greater than those in animals that received the diet containing RD and DDG (*p* < 0.05).

All blood parameter values were within the reference ranges: 85 to 150 mg/dL for glucose, 17.8 to 64.2 mg/dL for urea and 32 to 75 mg/dL for triglycerides [22].

## 4. Discussion

### 4.1. Digestible Contents

The variation in the chemical composition of the coproducts studied confirms the hypothesis that manufacturing processes influence nutritional characteristics. For most of the variables studied, the HPDDG showed better nutritional value than the other coproducts because the process of removing part of the fiber from the raw material generates a smaller particle size and concentrates lipids and proteins [23]. However, the coproducts DDG, CBS and DDGS generated similar results, mainly regarding the DE, DE:GE, ME and ME:GE values. Similar to the present study, nutritional variation between grain ethanol coproducts was also observed in the works by Cristobal et al. [24], Curry et al. [25] and Espinosa et al. [26], who observed that the ME, CP and EE digestibilities of the ingredients for pigs vary depending on the coproduct.

Variability in chemical composition has been demonstrated in studies that recorded mean values of 870 to 900 g/kg for DM, 40 to 50 g/kg for ash, 300 to 320 g/kg for CP, 90 to 110 g/kg for EE and 370 to 470 g/kg for NDF [7,20,27,28]. When performing a meta-analysis with 90 maize DDGS, Zeng et al. [29] recorded coefficients of variation of 25, 8, 36, 13 and 18% for ash, CP, EE, NDF and lysine, respectively.

In the present study, the greatest changes occurred in the CP, ash, EE and PS contents among the coproducts. In this sense, Böttger and Südekum [2] considered that the content and digestibility of protein and amino acids in DDGS are predetermined by the properties of the grain but are influenced by drying, heating and blending of product streams. Differences in the chemical composition of coproducts impact the utilization of coproducts and animal performance [24,30,31].

The HPDDG presented DE and ME values superior to those of the coproducts DDG, CBS and DDGS. This finding is related to the high levels of GE, EE and CP and lower PS in the HPDDG, which generated higher digestibility coefficients. The GE and NDF contents are key parameters for predicting the DE and ME of ethanol coproducts [27,32], as well as the EE [28].

The HPDDG energy values of this study were less than 4494 and 4555 kcal/kg DE for the two sources of HPDDG [33] and 4945 and 4669 kcal/kg for DE and ME in HPDDGS [24] but were higher than those recorded by Paula et al. [34], who recorded DE and ME values of 4399 and 4070 for HPDDG in Brazil and 3740 and 3477 kcal/kg for HPDDG in the USA, respectively. Yang et al. [35] recorded values equivalent to 3458 and 2268 kcal/kg DE and 3293 and 2168 kcal/kg ME for two HPDDGs produced in the USA.

The energy content of the coproducts of the present study differs from the classic reference of the NRC [36], which presents DE, ME and NE values of 4040, 3732 and 2342 kcal/kg for HPDDG; 3355, 3158 and 2109 kcal/kg for DDG; and 3582, 3396 and 2343 kcal/kg for DDGS with medium oil content, respectively. Variability in the nutritional composition, quality and process of obtaining coproducts can alter the energy value and contribute to differences in nutrient digestibility.

The energy levels of CBS were lower than those recorded by Anderson et al. [27], who recorded 3282 and 3031 kcal/kg of DE and ME, by Paula et al. [34], who recorded 3246 and 3060 kcal/kg of DE and ME, and by Yang et al. [35], who recorded 3231 and 3145 kcal/kg of DE and ME, when analyzing maize fiber and soluble coproducts, respectively.

The DE and ME values of the DDGS in this study are greater than the 3641 and 3417 kcal/kg of DE and ME recorded by Paula et al. [34]; greater than the 3231 and 3047 of DE and 3116 and 2880 of ME from two the DDGS sources investigated by Yang et al. [35]; and close to the highest values, from 3069 to 3363 kcal/kg of DE and 2769 to 3142 kcal/kg of ME, observed by Espinosa et al. [26] when analyzing eight DDGS with oscillation. Variations in energy content were also recorded by Anderson et al. [27], with 3841 to 4332 DE and 3414 to 4141 ME when evaluating seven origins of DDGS; by Li et al. [28], with 3255 to 4103 DE and 2960 to 3899 ME when evaluating 25 DDGS origins; and by Kerr et al. [7], with 3474 to 3807 DE and 3302 to 3603 ME when evaluating 11 sources of DDGS. These results suggest that, due to the wide ranges in chemical composition among corn coproducts, the use of clear definitions of these ingredients is necessary to minimize confusion in composition and feeding value for all animal species because they are produced by different processes [37].

The diets containing HPDDG stood out regarding the ATTD of the main organic contents (OM, EE, NDF) with the exception of CP, which is directly linked to the chemical composition of the coproduct. With a high lipid and protein content but low fiber content, the digestibility of HPDDG tends to be better than that of the other coproducts evaluated [23]. An increase in lipids in the diet improves the digestibility of other nutrients, increases the available energy content and reduces the caloric increment, as reviewed by Wealleans et al. [38]. In contrast, high levels of fibrous components can reduce nutrient digestibility and affect the digesta passage rate and energy metabolism in pigs, as reviewed by Li et al. [28]. In this sense, diets containing CBS stand out because of the high ATTD of EE but low ATTD of NDF. Studies have shown that the ATTD of nutrients is highly variable among coproduct sources with variable oil contents [7,26].

The results of the present study are consistent with those published by Paula et al. [35] when considering the ATTD values of DM, CP and NDF for HPDDG, which were higher than those for CBS and DDGS, but there was no difference in the ATTD values of EE between these coproducts. However, these values differ from those of Palowski et al. [4], who reported higher values of in vitro digestibility of CP for CBS than for HPDDG and DDGS, while HPDDG and CBS showed higher in vitro values of digestible NDF than DDGS.

### 4.2. Nitrogen Balance

With a higher CP content than the other coproducts, HPDDG generated greater N consumption and excretion, allowing greater amounts of retained N compared to CBS and DDGS but similar amounts to DDG. However, diets containing DDG, CBS or DDGS did not affect the N balance in the animals, despite having levels of 310, 140 and 280 g/kg of CP, respectively. The levels of lipids and fiber present in the coproducts can influence the digestibility of the diets and, consequently, the N balance [9]. These considerations are important because corn ethanol coproducts are used primarily as a source of protein for pigs.

The results of this study are consistent with those presented by Paula et al. [34], who reported greater N intake and excretion for diets containing HPDDG than for those containing CBS and DDGS. The N efficiency of retention of diets with different coproducts was similar to the values of 580 to 540 g/kg recorded by McDonnell et al. [39], who evaluated 0 to 300 g/kg DDGS in the diets; greater than those of Dahlen et al. [40] for DDG and DDGS; and lower than those indicated by Adeola and Kong [41], 580, 660 and 610 g/kg for DDGS of sorghum, triticale and corn, respectively.

### 4.3. Blood Parameters

Higher values of EE and PS and lower values of NDF and CP in coproducts suggest that they account for higher circulating levels of triglycerides in pigs fed CBS. However, animals fed with HPDDG had higher levels of circulating lipids only compared to those fed with DDG, suggesting the influence of the fiber. Serum triglycerides were increased in pigs fed sugar beet pulp compared with pigs fed diets containing no added fiber, while cholesterol was unaffected [42]. Pigs fed a high-fiber diet had lower total portal volatile fat acid flux [43]. Investigating the inclusion of glycerin in diets for piglets, Verussa et al. [21] reported no effect on cholesterol and triglyceride levels.

The urea levels in the pigs were directly related to the protein content of the coproducts. Diets containing coproducts with higher protein contents generated higher protein contents and higher N intake, which were converted into higher amounts of urea and excreted by the pigs. Higher urea levels were also recorded in pigs fed 300 g/kg DDGS than in those fed the control diet [43], while other studies did not observe a difference [21,44] or even higher urea levels in pigs fed the control diet [45].

Contradicting the hypothesis that corn ethanol coproducts could influence postfeeding glycemic levels, the present study did not observe changes in glucose levels, nor did other studies that investigated different fiber inclusions [42,44,45] or glycerin [21].

## 5. Conclusions

HPDDG, DDG, CBS and DDGS showed 4498, 3419, 3029 and 3335 kcal/kg DE; 4366, 3305, 2934 and 3214 kcal/kg ME; and 2515, 1938, 1649 and 1725 kcal/kg NE, respectively. Among the Brazilian coproducts, HPDDG had the highest energy levels and the best digestibility coefficients. The chemical composition of coproducts influences the N balance and circulating levels of urea and triglycerides in pigs.

## Figures and Tables

**Table 1:** Composition and calculated nutritional values of the reference diet (as-fed basis).

Item (g/kg)	RD	HPDDG	DDG	CBS	DDGS
Corn	636.4	509.12	509.12	509.12	509.12
Soybean meal	298.8	239.04	239.04	239.04	239.04
HPDDG	0.00	200.00	0.00	0.00	0.00
DDG	0.00	0.00	200.00	0.00	0.00
CBS	0.00	0.00	0.00	200.00	0.00
DDGS	0.00	0.00	0.00	0.00	200.00
Rice bran	30.0	24.00	24.00	24.00	24.00
Soybean oil	7.9	6.32	6.32	6.32	6.32
Limestone	6.6	5.28	5.28	5.28	5.28
Dicalcium phosphate	11.8	9.44	9.44	9.44	9.44
Vitamin–trace mineral premix ^1^	3.0	2.40	2.40	2.40	2.40
Salt	4.3	3.44	3.44	3.44	3.44
L-Lysine.HCl	0.9	0.72	0.72	0.72	0.72
DL-Methionine	0.3	0.24	0.24	0.24	0.24
Analyzed composition ^2^, g kg^−1^					
Metabolizable energy, kcal kg^−1^	3434	3450	3144	3040	3213
Dry matter	895.8	914.2	908.5	904.2	906.8
Crude protein	195.4	231.2	206.3	178.8	181.7
Total phosphorus	6.3	11.3	5.1	13.2	10.8
Calculated composition ^3^					
Calcium	6.5	-	-	-	-
Sodium	1.8	-	-	-	-
Digestible lysine	9.7	-	-	-	-

RD = reference diet; HPDDG = high-protein distiller’s dried grain; DDG = distiller’s dried grain; CBS = corn bran with solubles; DDGS = distiller’s dried grain with solubles; ^1^ Provided per kg of diet: Vitamin A (retinyl acetate) 13,750 IU, Vitamin B_1_ (thiamine) 2 mg, Vitamin B_2_ (riboflavin) 1.25 mg, Vitamin B_3_ (niacin) 50 mg, Vitamin B_5_ (D-pantothenic acid) 30 mg, Vitamin B_6_ (pyridoxine-HCl) 4 mg, Vitamin B_9_ (folic acid) 0.625 mg, Vitamin B_12_ (cyanocobalamine) 4.5 μg, Vitamin D_3_ (cholecalciferol) 3000 IU, Vitamin E (dl-α-tocopherol acetate) 75 IU, Vitamin K_3_ (menadione) 6.25 mg, cobalt (CoSO_4_·H_2_O) 1.25 mg, copper (CuSO_4_·5H_2_O) 25 mg, iron (FeSO_4_·H_2_O) 150 mg, zinc (ZnO) 200 mg, manganese (MnO) 75 mg, selenium (Na_2_O_3_Se) 0.7 mg, iodine (Ca(IO_3_)_2_) 2 mg, choline chloride (250 mg) and biotin (25 μg). ^2^ Methodology described by AOAC [16]. ^3^ Based on feedstuff composition report by Rostagno et al. [15].

**Table 2:** Chemical composition of ethanol corn coproducts (dry matter basis).

Item	HPDDG	DDG	CBS	DDGS
Gross energy (kcal/kg)	5186	4798	4206	4465
Dry matter (g/kg)	914.1	909.7	893.2	882.1
Organic matter (g/kg)	885.6	893.8	850.5	868.7
Crude protein (g/kg)	405.8	312.4	140.7	279.5
Ether extract (g/kg)	167.1	66.8	94.1	82.5
Neutral detergente fiber (g/kg)	483.4	534.4	464.4	542.7
Ash (g/kg)	28.5	16.0	42.7	13.4
Particle size (μm)	485	653	1107	672

HPDDG = high-protein distiller’s dried grain; DDG = distiller’s dried grains; CBS = corn bran with solubles; DDGS = distiller’s dried grains with solubles.

**Table 3:** Energy and digestibility coefficients of diets and ethanol corn coproducts.

Item	RD	HPDDG	DDG	CBS	DDGS	SEM	*p*-Value
	Diets						
DM intake (kcal/d)	1328	1341	1318	1314	1342	-	-
GE (kcal/kg)	3900	4194	4061	4007	4027	-	-
DE (kcal/kg)	3459 ^ab^	3667 ^a^	3451 ^b^	3373 ^b^	3438 ^b^	27.54	0.006
ME (kcal/kg)	3419 ^ab^	3609 ^a^	3397 ^ab^	3322 ^b^	3378 ^b^	27.89	0.011
DE:GE	876.7	873.3	848.3	841.7	853.3	0.01	0.112
ME:GE	876.7	860.0	836.7	826.7	840.0	0.01	0.083
	Coproducts						
DE:GE	-	0.86 ^a^	0.71 ^b^	0.72 ^b^	0.75 ^ab^	0.20	0.011
ME:GE	-	0.84 ^a^	0.69 ^b^	0.69 ^b^	0.72 ^ab^	0.20	0.015
NE:GE	-	0.48 ^a^	0.40 ^b^	0.39 ^b^	0.39 ^b^	0.90	0.001

RD = reference diet; HPDDG = high-protein distiller’s dried grain; DDG = distiller’s dried grains; CBS = corn bran with solubles; DDGS = distiller’s dried grains with solubles; SEM = standard error of the mean; DM = dry matter; GE = gross energy; DE = digestible energy; ME = metabolizable energy; NE = net energy. ^a,b^ Means followed by different letters on the line differ statistically at 5% probability according to the Tukey test.

**Table 4:** Digestible contents and digestibility coefficients of diets with ethanol corn coproducts.

Item	RD	HPDDG	DDG	CBS	DDGS	SEM	*p*-Value
	Digestible contents (g/kg)						
Dry matter	707.5	725.3	690.9	678.8	693.9	5.71	0.097
Organic matter	819.6 ^b^	841.9 ^a^	824.7 ^ab^	817.6 ^b^	818.2 ^b^	1.96	0.002
Crude protein	149.1 ^bc^	179.1 ^a^	158.1 ^b^	134.9 ^c^	134.8 ^c^	6.43	<0.001
Ether extract	16.9 ^b^	40.0 ^a^	14.1 ^b^	35.3 ^a^	18.8 ^b^	22.32	<0.001
Neutral detergent Fiber	158.5 ^b^	213.3 ^a^	218.2 ^a^	141.5 ^b^	167.9 ^b^	16.56	<0.001
Ash	24.8	22.6	21.8	28.4	28.4	15.52	0.129
	Digestibility coefficients						
Dry matter	0.79	0.79	0.76	0.75	0.76	0.06	0.072
Organic matter	0.97 ^a^	0.97 ^a^	0.95 ^b^	0.96 ^ab^	0.95 ^b^	0.03	0.003
Crude protein	0.76	0.77	0.77	0.75	0.74	0.04	0.585
Ether extract	0.53 ^ab^	0.64 ^a^	0.46 ^b^	0.65 ^a^	0.54 ^ab^	0.02	0.003
Neutral detergent Fiber	0.68 ^ab^	0.75 ^a^	0.66 ^b^	0.53 ^c^	0.59 ^bc^	0.07	<0.001
Ash	0.51	0.52	0.53	0.57	0.60	0.01	0.353

RD = reference diet; HPDDG = high-protein distiller’s dried grain; DDG = distiller’s dried grains; CBS = corn bran with solubles; DDGS = distiller’s dried grains with solubles; SEM = standard error of the mean. ^a,b,c^ Means followed by different letters on the line differ statistically at 5% probability according to the Tukey test.

**Table 5:** Nitrogen (N) balance of pigs fed diets with ethanol corn coproducts.

Item	RD	HPDDG	DDG	CBS	DDGS	SEM	*p*-Value
N intake (g/d)	41.27 ^b^	49.44 ^a^	43.20 ^ab^	37.41 ^b^	38.82 ^b^	14.92	0.001
Fecal N (g/d)	9.93	10.99	10.02	9.12	9.97	3.69	0.460
Urine N (g/d)	6.26	10.03	9.59	6.82	9.64	6.31	0.110
N excreted (g/d)	16.19 ^ab^	21.02 ^a^	19.62 ^ab^	15.94 ^b^	19.62 ^ab^	7.81	0.019
N retained (g/d)	25.07 ^ab^	28.42 ^a^	23.59 ^ab^	21.47 ^b^	19.20 ^b^	9.80	0.001
Efficiency (%) ^1^	60.75 ^a^	57.48 ^a^	54.61 ^ab^	56.78 ^a^	49.46 ^b^	0.11	0.018

RD = reference diet; HPDDG = high-protein distiller’s dried grain; DDG = distiller’s dried grains; CBS = corn bran with solubles; DDGS = distiller’s dried grains with solubles; SEM = standard error of the mean. ^1^ Efficiency = N retained:N intake; ^a,b^ Means followed by different letters on the line differ statistically at 5% probability according to the Tukey test.

**Table 6:** Serum biochemical indicators of pigs fed diets containing ethanol corn coproducts.

Item	RD	HPDDG	DDG	CBS	DDGS	SEM	*p*-Value
Glucose (mg/dL)	81.87	78.17	78.00	69.17	83.17	2.14	0.298
Urea (mg/dL)	18.17 ^b^	29.83 ^a^	25.33 ^ab^	19.67 ^b^	27.83 ^ab^	1.27	0.007
Triglycerides (mg/dL)	35.33 ^cd^	51.00 ^b^	33.33 ^d^	66.33 ^a^	43.67 ^bc^	2.45	<0.001

RD = reference diet; HPDDG = high-protein distiller’s dried grain; DDG = distiller’s dried grains; CBS = corn bran with solubles; DDGS = distiller’s dried grains with solubles; SEM = standard error of the mean. ^a–d^ Means followed by different letters on the line differ statistically at 5% probability according to the Tukey test.

## Data Availability

The original contributions presented in the study are included in the article, further inquiries can be directed to the corresponding author.
